# Suprapatellar vs infrapatellar approaches for intramedullary nailing of distal tibial fractures: a systematic review and meta-analysis

**DOI:** 10.1186/s10195-023-00694-7

**Published:** 2023-04-11

**Authors:** Chen-Yuan Yang, Soon-Tzeh Tay, Liang-Tseng Kuo

**Affiliations:** 1 Department of Orthopedics, Kuang Tien General Hospital, Taichung, 433 Taiwan; 2 Department of Nursing, Hungkuang University, Taichung, 433 Taiwan; 3 School of Medicine, Chang Gung University, Taoyuan, 333 Taiwan; 4 Department of Orthopedic Surgery, Chang Gung Memorial Hospital, No. 6 Western Sec., Chia-Pu Road, Putzi City, Chiayi, 613 Taiwan

**Keywords:** Suprapatellar, Infrapatellar, Tibia fracture, Intramedullary nailing, Distal tibia fracture

## Abstract

**Background:**

This review was conducted to compare the efficacy of suprapatellar (SP) and infrapatellar (IP) approaches for treating distal tibial fractures with intramedullary nailing.

**Method:**

This systematic review included studies comparing the outcomes of patients receiving nailing for distal tibial fractures using the SP and IP approaches. We searched the Cochrane CENTRAL, MEDLINE and Embase databases for relevant studies till 18th Sep. 2022. We used the Newcastle Ottawa Scale to assess study quality and a random-effects meta-analysis to synthesize the outcomes. We used the mean difference (MD) or standardized mean difference (SMD) with the 95% confidence interval (CI) for continuous data and the odds ratio (OR) with the 95% CI for dichotomous data.

**Results:**

Four studies with 586 patients (302 in the SP group and 284 in the IP group) were included in this systematic review. The SP group may have had little or no difference in pain and slightly better knee function (MD 3.90 points, 95% CI 0.83 to 5.36) and better ankle function (MD: 8.25 points, 95% CI 3.35 to 13.15) than the IP group 12 months after surgery. Furthermore, compared to the IP group, the SP group had a lower risk of malalignment (OR: 0.22, 95% CI 0.06 to 0.75; number needed to treat (NNT): 6), a lower risk for open reduction (OR: 0.58, 95% CI 0.35 to 0.97; NNT: 16) and a shorter surgical time (MD: − 15.14 min, 95% CI − 21.28 to − 9.00).

**Conclusions:**

With more advantages, the suprapatellar approach may be the preferred nailing technique over the infrapatellar approach when treating distal tibial fractures.

*Level of evidence:* Level III, systematic review of non-randomized studies.

**Supplementary Information:**

The online version contains supplementary material available at 10.1186/s10195-023-00694-7.

## Introduction

Distal tibia fractures mainly result from torsional or axial loading injuries; the fracture patterns are usually spiral or comminuted, necessitating surgical intervention to achieve proper reduction and stable fixation. The surgical treatment options include external fixation, plate fixation and intramedullary nails. However, the easily compromised soft tissue envelope, associated swelling and open wounds make it more challenging to manage distal tibial fractures without complications.

While plate fixation can achieve better alignment control than an intramedullary nail, related soft tissue complications remain unavoidable despite the use of the minimally invasive plate osteosynthesis (MIPO) technique [1]. Intramedullary nail fixation is a minimally invasive procedure with the lowest damage to soft tissue. However, the traditional infrapatellar approach requires the knee to be in a flexion position during the nailing procedure, from entry point creation to reaming and implant insertion, making it challenging to control the alignment and fix the nail correctly in the wide canal of the distal tibial fragment [2]. Recently, suprapatellar intramedullary nails have become more widely accepted by orthopaedic surgeons because of the advantages of easier fracture reduction control and the facilitation of fluoroscopic images in the semi-extended position.

However, the optimal surgical approach to nailing for the treatment of distal tibial fractures is still a subject of debate, despite a range of studies comparing outcomes between the two approaches [3–5]. We, therefore, conducted this systematic review to compare the efficacies, with regard to radiological and functional outcomes, of the suprapatellar and infrapatellar approaches when treating distal tibia fractures by intramedullary nailing.

## Methods

### Search strategy and study selection

We followed the Preferred Reporting Items for Systematic Reviews and Meta-Analyses (PRISMA) reporting guidelines [6] and the recommendations of the Cochrane Collaboration to conduct this systematic review and meta-analysis [7]. We registered the protocol of this systematic review in PROSPERO (CRD42022383970). We searched online databases, including MEDLINE, Embase, Cochrane CENTRAL and other sources, using the keywords: “distal tibia”, “fracture”, and “nail” for all eligible studies up to 18 September 2022. The details of the search strategy are listed in Additional file 1 of Appendix 1. In addition, we also checked the reference lists of potentially eligible studies for further relevant studies. We also searched the trial register (www.clinicaltrials.gov) for ongoing trials and consulted experts in this field for any ongoing studies. We applied no language limitations.

### Inclusion and exclusion criteria

We aimed to include randomized controlled trials (RCTs) or non-randomized comparative studies investigating the outcomes of suprapatellar and infrapatellar nailing for the treatment of distal metaphyseal fracture in this study. Study designs with a single cohort and without a comparison group, review studies and case series were excluded. Non-human studies were also excluded.

Two reviewers (CYY and LTK) independently identified potential studies. First, we excluded irrelevant studies after going through the titles and abstracts. Second, we checked the full texts of potential studies against the inclusion/exclusion criteria. Third, any discrepancies between the reviewers’ assessments were resolved through discussion, with a third reviewer consulted in the case of disagreement.

### Quality assessment

We used the Newcastle–Ottawa Scale (NOS) to assess the quality of non-randomized studies [8]. Appraisal criteria included participant selection, comparability and outcomes. The same two reviewers independently assessed the included studies, and a third reviewer was consulted in the case of disagreement.

### Data extraction

Data were extracted from the included studies by two independent authors using the predesigned data tables. Relevant data included patient characteristics, sample size, surgical technique, duration of follow-up and outcomes. The outcomes included pain level, ankle functional assessment and radiographic outcomes. We contacted the relevant authors for more information if the data could not be extracted directly from the original studies. The primary outcomes were pain levels and functionality, and the secondary outcomes were radiologic deviation angle in the coronal and sagittal planes, malalignment, the need for open reduction, surgical time and complications. If outcomes were reported at more than one time point, we preferred to extract data 1 year after the surgery.

### Data analysis

This study used Review Manager 5.4 software (Review Manager Version 5.1.6, Copenhagen: The Nordic Cochrane Centre, The Cochrane Collaboration, 2020) for the meta-analysis. We used random-effects meta-analysis due to the inherent clinical heterogeneity of the included studies [9]. We used the risk difference (RD) with the 95% confidence interval (CI) for binary outcomes to report the meta-analysis outcomes. We calculated the number needed to treat (NNT) or the number needed to harm (NNTH) to better understand the magnitude of the effect estimate if the outcome showed a statistical significance. For dichotomous outcomes, such as treatment success or adverse events, we calculated the NNT or the NNTH from the control group event rate and the risk ratio using the Cates Visual Rx NNT Calculator [10].

We used the mean difference (MD) with the 95% CI for continuous outcomes. A *P* value of < 0.05 was set as the threshold of statistical significance. We used *X*^2^ and *I*^*2*^ statistics to assess statistical heterogeneity, with a *P* value of < 0.10.* I*^*2*^ values of 0–24.9%, 25–49.9%, 50–74.9% and 75–100% were set to indicate no, low, moderate and high heterogeneity, respectively [11]. We planned to perform subgroup analyses when there was significant or clinical heterogeneity if there were sufficient studies. We also estimated the inter-study variance using tau-squared (*τ*^2^) statistics [11].

## Results

### Included studies

**Fig. 1:**
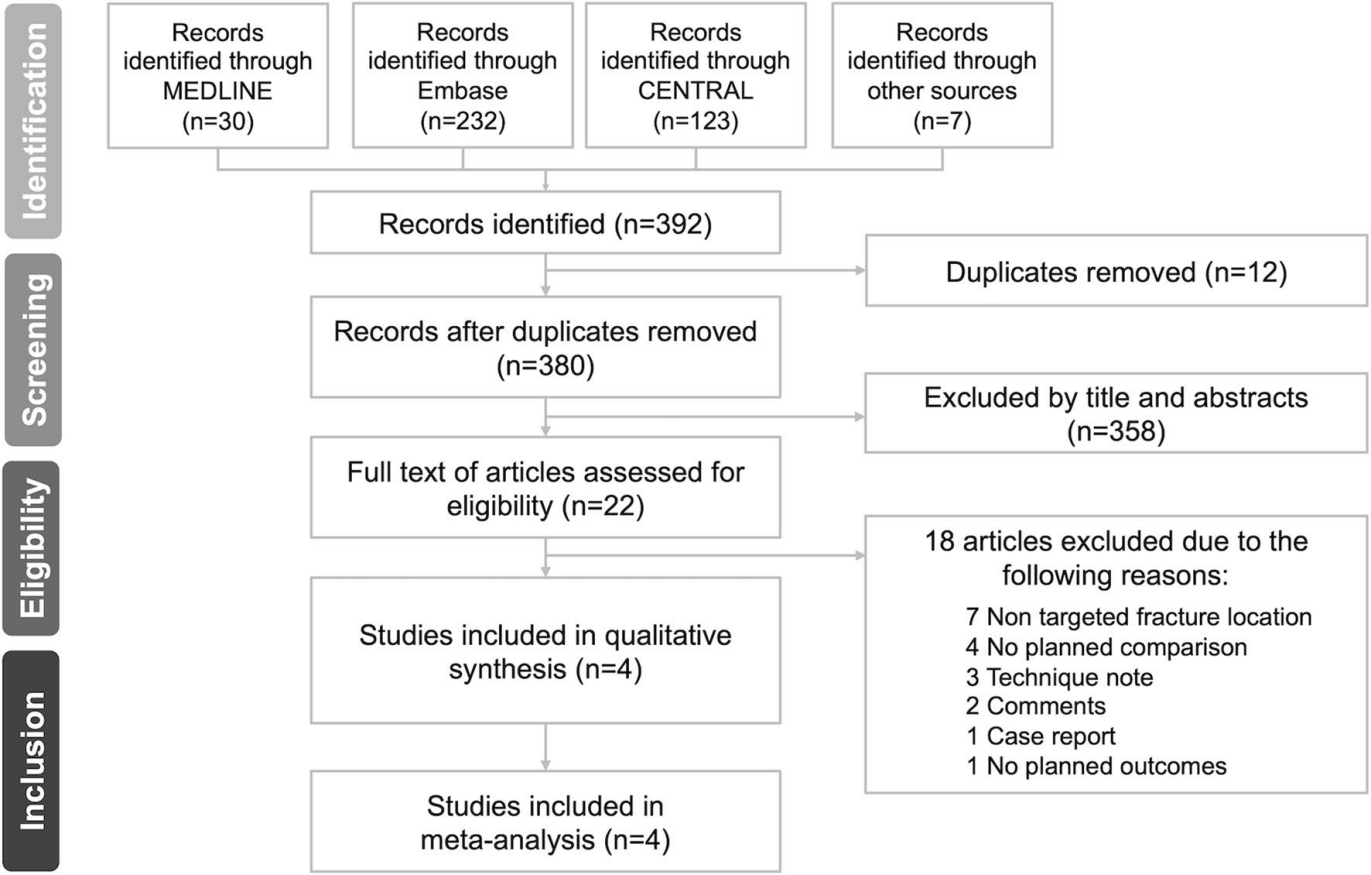
PRISMA flow diagram of the study

After searching the above databases for all existing studies, we identified 30 records from MEDLINE, 232 from Embase and 123 from CENTRAL. Two additional records were identified from the reference lists of the eligible studies, and five ongoing trials were identified after searching the trial registers and consulting specialists. A total of 392 records were identified, from which 12 duplicates were removed. After screening all the records by titles and abstracts, 358 were excluded. We assessed the remaining 22 full-text articles and excluded 18 records for the below-stated reasons (Fig. 1, Additional file 1 in Appendix 2). Ultimately, four studies were included in the qualitative synthesis and meta-analysis [2–5].

### Study characteristics

The qualitative systematic review included four studies with 586 patients (302 in the suprapatellar nailing group and 284 in the infrapatellar nailing group) (Table 1) [2–5]. The included studies were published between 2016 and 2022, and the enrolled sample sizes ranged from 63 to 266. All the included studies compared the outcomes between suprapatellar and infrapatellar intramedullary nailing for distal tibial fractures. All included studies were retrospective cohort studies. The mean age for the two groups in each study was around 30 to 40 years. The follow-up period in these studies was at least 1 year. The outcomes and measurements included pain severity measured with the visual analogue scale (VAS), knee function measured by the Lysholm score, ankle function measured by the American Orthopedic Foot and Ankle Society (AOFAS) score, surgical times, the need for open reduction, and radiologic outcomes, including coronal and sagittal angulations and malalignment rates. The details of the included studies are shown in Table 1.

**Table 1:** Study characteristics

Author, year	Study design	Group	No. of patients	Age (years)	Gender male (%)	Follow-up (months)	Outcome	Quality
Avilucea et al. 2016 [2]	RCS	SP	132	33.6	88 (66.7)	N/A	1°: N/A	8
		IP	134	35.4	76 (56.7)		2°: Radiologic/open reduction	
Lu et al. 2020 [3]	RCS	SP	27	42.6	11 (40.7)	23.2	1°: VAS/Lysholm score/AOFAS score 2°: Radiologic outcomes/need for open reduction/surgical time	8
		IP	36	40.6	20 (55.6)	24.3		
Hague et al. 2021 [4]	RCS	SP	74	41.3	56 (76.6)	N/A	1°: N/A	7
		IP	51	39.3	38 (75.4)		2°: Radiologic outcomes	
Gao et al. 2022 [5]	RCS	SP	69	45.6	49 (71.0)	14.2	1°: VAS/Lysholm score/AOFAS score	8
		IP	63	44.1	38 (60.3)		2°: Radiologic outcomes/need for open reduction/surgical time	

*AOFAS* American Orthopedic Foot and Ankle Society, *IP* infrapatellar, *N/A* not available,* No*. number, *RCS* retrospective cohort study, *SP* suprapatellar, *VAS* visual analogue scale

### Fracture patterns and details of the surgery

The details regarding fracture patterns and surgical details are shown in Tables 2 and 3. Three of the included studies used the Müller Arbeitsgemeinschaft für Osteosynthesefragen/Orthopaedic Trauma Association (AO/OTA) fracture classification [12]. The AO/OTA classification describes the fracture in defined terminology according to its location, segment, and complexity (intraarticular or extraarticular). All patients in two of the studies had type 43A fractures (distal tibial extraarticular fractures) only [4, 5], whereas another study enrolled patients with fracture types 43 A/C1/C2 (43C1: simple articular, simple metaphyseal, distal tibial fracture; 43C2: simple articular, multifragmentary metaphyseal, distal tibial fracture) [3]. Concerning fracture location, two studies measured the fracture distance to the tibial plafond [2, 5], one study enrolled fractures within two Müller squares [4], and the fourth study included participants with fractures between the shaft and plafond [3]. Avilucea et al. [2] and Hague et al. [4] reported the ratio of open fractures and concomitant fibular fractures, whereby the incidence of open fractures ranged from 22.2% to 49.6%. The incidence of concomitant fibular fracture ranged from 88.0% to 94.7%, and adjunctive fibular fixation was needed by 3–20%.

**Table 2:** Fracture characteristics and surgical details

Author, year	Group	No. of patients	Fracture condition				Surgical details	
			OTA classification (43-A/C1/C2)	Distance from fracture to plafond (mm)	Open fracture *N* (%)	Fibular fracture *N* (%)	Fibular fixation *N* (%)	Need for open reduction *N* (%)
Avilucea et al. 2016 [2]	SP	132	N/A	42.3 ± 3.1	28 (21.2)	126 (95.5)	22 (16.7)	18 (13.6)
	IP	134	N/A	41.4 ± 3.3	31 (23.1)	125 (93.3)	32 (23.9)	23 (17.2)
Lu et al. 2020 [3]	SP	27	15/8/4	< 120	N/A	N/A	N/A	2 (7.4)
	IP	36	20/9/7	< 120	N/A	N/A	N/A	6 (16.7)
Hague et al. 2021 [4]	SP	74	74/0/0	< Two Müller squares	44 (59.5)	67 (90.5)	4 (6.0)	N/A
	IP	51	51/0/0	< Two Müller squares	18 (35.3)	43 (84.3)	0 (0.0)	N/A
Gao et al. 2022 [5]	SP	69	69/0/0	40.1 ± 5.2	N/A	N/A	N/A	8 (11.6)
	IP	63	63/0/0	41.0 ± 6.8	N/A	N/A	N/A	16 (25.4)

*IP* infrapatellar, *N/A* not available, *No*. number, *OTA* Orthopaedic Trauma Association, *SP* suprapatellar

**Table 3:** Summary of findings

Outcome	*N*	Participants (SP/IP)	Overall effect		Heterogeneity	
			MD/RR/SMD (95% CI)	*P*	*I*^2^ %	*P*
Pain level measured by VAS (12 months after surgery)						
All included studies	2	96/99	− 0.91 (− 3.21, 1.39)	0.44	98	< 0.001
Lysholm score (12 months after surgery)						
All included studies	2	96/99	3.09 (0.83, 5.36)	0.007	0	0.83
AOFAS score (12 months after surgery)						
All included studies	2	96/99	8.25 (3.35, 13.15)	0.001	91	< 0.001
Radiologic outcomes						
AP angulation (°)	3	275/248	− 2.09 (− 2.47, − 1.71)	< 0.001	75	0.02
LAT angulation (°)	3	275/248	− 1.40 (− 3.43, 0.64)	0.18	99	< 0.001
Malalignment	4	302/284	0.22 (0.06 to 0.75)	0.02	77	0.004
Surgical details						
Need for open reduction	3	228/233	0.58 (0.35, 0.97)	0.04	0	0.46
Surgical time	2	96/99	− 15.15 (− 21.28, − 9.00)	< 0.001	59	0.12

*AOFAS* American Orthopedic Foot and Ankle Society, *AP* anteroposterior,* IP* infrapatellar nailing, *LAT* lateral, *MD* mean difference, *RR* risk ratio,* SMD* standardized mean difference, *SP* suprapatellar nailing

### Study quality

We used the NOS scale to assess the quality of the four included studies. All four studies achieved 4 points for proper selection of the intervention and control cohorts. Only one study showed considerable risk in comparability on account of the baseline inequality between the two groups [4]. All four studies scored well in outcome domains. Details of the quality assessment of the studies can be accessed in Additional file 1 in Appendix 3.

### Primary outcomes

#### Pain level at 1 year following surgery

**Fig. 2:**
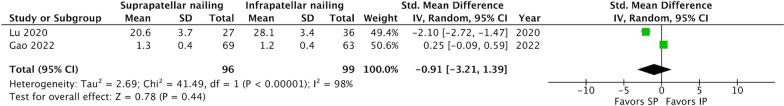
Forest plot of pain at 1 year after surgery. There was no significant difference between the two groups concerning the level of pain reduction (SMD: − 0.92, 95% CI − 3.19 to 1.35; *P* = 0.43; *I*^2^ = 98%). *MD* median difference, *CI* confidence interval

Two of the included studies, comprising 195 patients, reported pain levels measured by VAS 1 year after surgery [3, 5]. However, the meta-analysis revealed no significant difference between the two groups with regard to pain level (SMD: − 0.91, 95% CI − 3.21 to 1.39; *P* = 0.44; *I*^2^ = 98%, Fig. 2). We therefore could not conclude whether the SP or IP approach led to better pain reduction in patients with distal tibial fractures. The sumamry of primary outcomes are provided in Table 3.

#### Function 1 year after surgery

##### Knee function

**Fig. 3:**
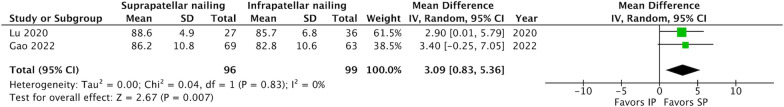
Forest plot of knee function by Lysholm score 12 months after surgery. The SP group had a higher Lysholm score than the IP group (MD: 3.90 points; 95% CI 0.83 to 5.36; *P* = 0.007; *I*^2^ = 0%). *MD* median difference, *CI* confidence interval

Two studies, comprising 195 patients, reported knee function at 1 year using Lysholm scores [3, 5]. The SP group had a higher Lysholm score than the IP group (MD: 3.90; 95% CI − 0.83 to 5.36; *P* = 0.007; *I*^2^ = 0%, Fig. 3). However, the magnitude of the difference did not reach the minimal clinically important difference (MCID) in the Lysholm knee score [13]. Therefore, we could not conclude that the SP approach afforded better knee function 1 year after the index surgery when compared with the IP approach.

##### Ankle function

**Fig. 4:**
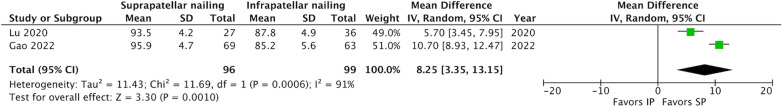
Forest plot of ankle function assessed by the AOFAS score 12 months after surgery. The SP group had a clinically significantly higher AOFAS score than the IP group (MD: 8.25 points; 95% CI 3.35 to 13.15; *P* = 0.001; *I*^2^ = 91%).* AOFAS* American Orthopedic Foot and Ankle Society,* MD* median difference,* CI* confidence interval

Two included studies, comprising 195 patients, reported ankle function as assessed by the AOFAS score [3, 5]. The SP group had a higher AOFAS score than the IP group (MD: 8.25 points; 95% CI 3.35 to 13.15; *P* = 0.001; *I*^2^ = 91%, Fig. 4). This difference was also clinically significant [14]. We therefore concluded that the SP approach offered clinically better ankle function 1 year after the distal tibial fracture surgery than the IP approach.

### Secondary outcomes

#### Degrees of angulation

**Fig. 5:**
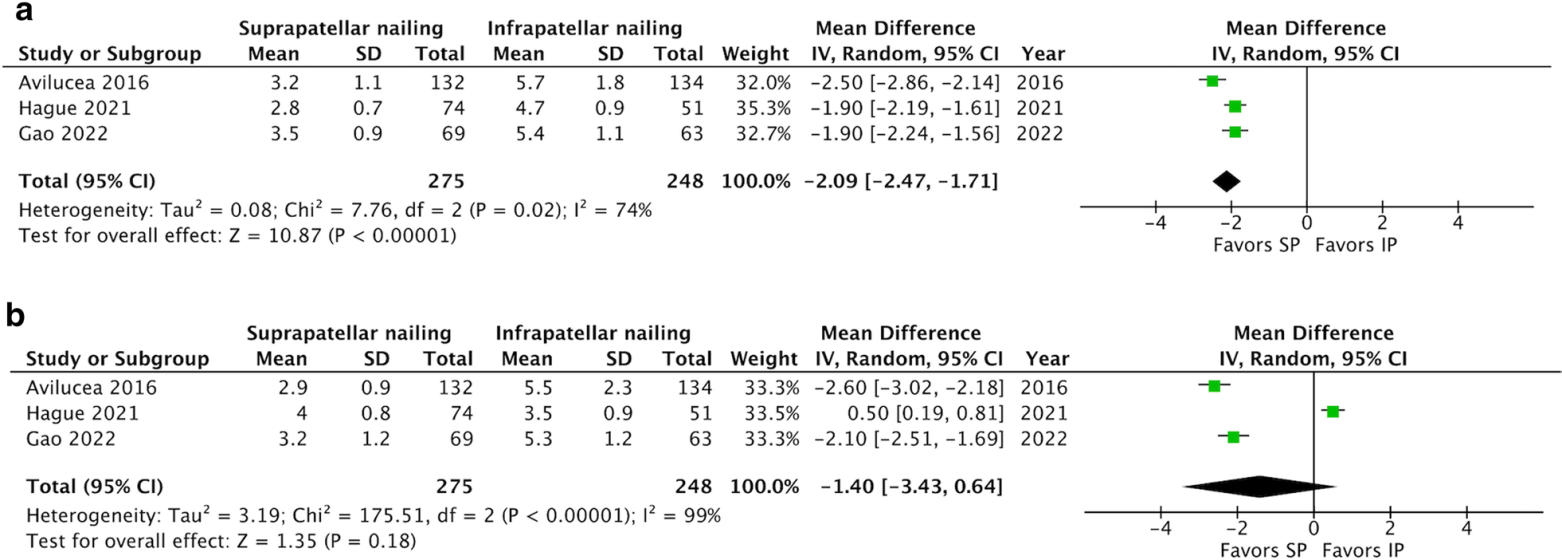
Forest plots of **a** coronal angulation and **b** sagittal angulation. Compared to SP, IP had greater coronal angulation (MD: − 2.09°; 95% CI − 2.47 to − 1.71; *P* < 0.001; *I*^2^ = 75%) but similar sagittal angulation (MD: − 1.40°; 95% CI − 3.43 to 0.64; *P* = 0.18; *I*^2^ = 99%).* CI *confidence interval, *MD* mean difference

Three of the included studies, comprising 523 patients, assessed the radiologic outcome following surgery [2, 4, 5]. All used a digital imaging system. As regards coronal alignment, the result of the meta-analysis revealed that IP had more degrees of coronal angulation than SP (MD: − 2.09°, 95% CI − 2.47 to − 1.71; *P * < 0.001; *I*^2^ = 75%, Fig. 5a). However, as regards sagittal alignment, there was no significant difference between the two groups in the degrees of sagittal angulation (MD: − 1.40°, 95% CI − 3.43 to 0.64; *P* = 0.18; *I*^2^ = 99%, Fig. 5b). We therefore concluded that SP provided a better outcome in regard to coronal angulation than IP. The sumamry of secondary outcomes are provided in Table 3.

#### Malalignment

**Fig. 6:**
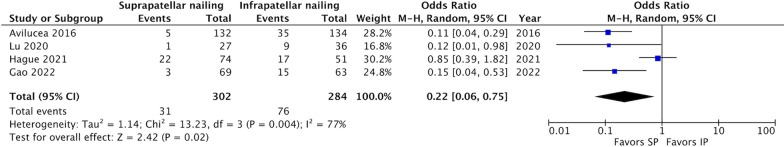
Forest plot of the malalignment. The SP group had a lower risk for postoperative malalignment than the IP group (OR: 0.22; 95% CI 0.06 to 0.75; NNT: 6 (95% CI 5 to 20); *P* = 0.02; *I*^2^ = 77%). *CI* confidence interval, *NNT* number needed to treat, *OR* odds ratio

All of the included studies, comprising 596 patients, assessed the radiologic alignment following surgery [2–5]. Three included studies defined malalignment as > 5° of angulation in the coronal or sagittal plane. One study reported coronal and sagittal malalignment separately. After a discussion, we chose the worst scenario for each group and subjected it to meta-analysis. The meta-analysis showed that SP nailing had a lower risk of postoperative malalignment than IP nailing (OR: 0.22, 95% CI 0.06 to 0.75; *P* = 0.02; *I*^2^ = 77%, Fig. 6). Taking the IP group with a risk of malalignment of 26.1% as the reference, the NNT for malalignment was 6 (95% CI 5 to 20) [2].

#### Surgical details

**Fig. 7:**
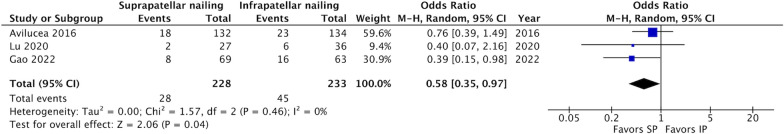
Forest plot of the need for open reduction. The SP group had a lower risk for open reduction than the IP group (OR: 0.58; 95% CI 0.35 to 0.97; NNT: 16 (95% CI 10 to 233); *P* = 0.04; *I*^2^ = 0%).* CI* confidence interval,* NNT* number needed to treat,* OR* odds ratio

**Fig. 8:**
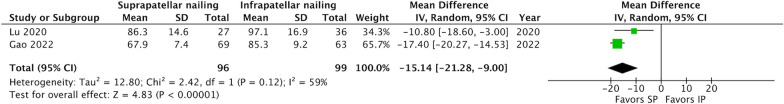
Forest plot of the surgical time. The SP group had a shorter surgical time than the IP group (MD: − 15.14 min; 95% CI − 21.28 to − 9.00; *P* < 0.001; *I*^2^ = 59%).* CI* confidence interval;* MD* mean difference

Three of the included studies, comprising 461 patients, reported the need for open reduction [2, 3, 5]. The pooled result revealed that SP nailing had a lower risk of the need for open reduction than IP nailing (OR: 0.58, 95% CI 0.35 to 0.97; *P* = 0.04; *I*^2^ = 0%, Fig. 7). The NNT of the need for open reduction was 16 (95% CI: 10 to 233), taking the IP group with 17.2% as the reference [2]. One episode of open reduction could be prevented for every 16 patients treated with SP nailing. Fracture reduction seemed easier in the SP group than in the IP group. Hence, the SP group also had a shorter surgical time than the IP group (MD: − 15.14 min, 95% CI − 21.28 to − 9.00; *P* < 0.001; *I*^2^ = 59%, Fig. 8).

### Complications

None of the included studies reported this outcome.

### Subgroup analysis

Due to the limited study number, we were unable to perform subgroup analyses.

## Discussion

In this meta-analysis comparing the efficacies of the two approaches for intramedullary nail fixation of distal tibia fractures, the suprapatellar group showed superiority in postoperative knee functional score, ankle function, coronal angulation and malalignment rate. Furthermore, the suprapatellar group had less need for open reduction and hence a shorter operation time than the infrapatellar group. The postoperative pain and sagittal angulation were similar in both the suprapatellar and infrapatellar groups. The functional and radiographic outcomes demonstrated that the suprapatellar approach was a more reasonable option than the infrapatellar approach for treating a distal tibia fracture with an intramedullary nail.

In contrast to other meta-analyses comparing the SP and IP approaches for tibia fracture, which indicated significant pain reduction in the SP group [15–17], the postoperative pain scores in our study did not show a significant difference between the SP and IP groups; however, a trend for reduced pain in the SP group was noticeable. Why the VAS data did not significantly differ between the two groups, while the AOFAS pain score of the SP group was significantly better in the Gao et al.’s enrolled study [5], is not clearly explained. Considering that knee pain could also be less after the SP approach than after the IP approach [18], the SP group should have a lower overall postoperative pain score. Otherwise, the longer follow-up time and mixed pain sources from the fracture site and the knee joint may have affected the VAS difference between these two groups. Results from more large-scale prospective studies are needed to support this hypothesis on postoperative pain.

The superior Lysholm scores in the suprapatellar group are consistent with a previous meta-analysis comparing the SP and IP approaches for tibia shaft intramedullary nailing [15, 17, 19]. The difference in Lysholm score might result from anterior knee pain, which could be significantly reduced using the SP approach [5, 18]. Up to 71% of patients receiving IP nailing complain of anterior knee pain, especially in the kneeling position, possibly due to patellar tendon or infrapatellar nerve violation, no matter whether the paratendinous or transtendinous method is used [20]. Although the reamers repeatedly go back and forth within the patellofemoral joint during the SP procedure, carrying risks of cartilage damage [21], the related complications were not reported in our enrolled studies and should be preventable by using a protection sleeve. Chan et al. [22] reported that three out of 11 patients had patellofemoral articular changes found by arthroscopic exam immediately after nailing, but none experienced related joint pain at 12 months follow-up. Umur et al. [23] performed a case–control study showing that the SP group had no postoperative anterior knee pain and a better Lysholm score than the IP group, despite two cases of patellofemoral cartilage degeneration detected by MRI. Another concern about knee function impairment is knee joint infection after SP nailing for tibia open-fracture fixation. Nonetheless, no knee sepsis complications were reported in a case series with 139 open tibia fracture patients undergoing SP nailing [24].

Ankle function can be influenced by fracture location, fracture comminution, articular involvement, soft tissue conditions (swelling, open wound, contamination), reduction quality, joint stiffness and post-traumatic arthritis. Two of the enrolled studies had fracture sites only 43 mm from the ankle joint [2, 5], and more than 40% of the cases in another study had intra-articular distal tibia fractures in each group [3]. Previous meta-analyses comparing SP and IP mainly focused on general tibia fracture or compared IP intramedullary nail with plate fixation for distal tibia fracture [25]. The clinical importance of the SP approach for distal tibia fracture was not demonstrated. Our data proved that the SP approach was significantly better than the IP approach as regards ankle function, and the change in AOFAS score (MD: 8.25; 95% CI: 3.35 to 13.15) was meaningful in regard to the minimal clinically important difference (MCID). The improvement in ankle function might correlate with the significantly reduced malalignment rate in the SP group. Ankle joint post-traumatic arthritis, even with deteriorated function, should be observed in the long-term follow-up. Furthermore, open fracture and soft tissue conditions were not compared, although these might also interfere with ankle function to some degree.

As regards the radiological result, the SP group showed a favourable result in coronal angulation and malalignment rate. However, sagittal angulation did not differ significantly between the two groups. Compared to tibia shaft fracture, distal tibia fractures are usually unstable because of long, spiral or comminuted patterns with concomitant fibula fracture, making it more difficult to postoperatively achieve proper alignment of IM nailing. At the same time, the width of the distal tibia metaphysis is much greater in the coronal plane than in the sagittal plane; therefore, coronal malreduction could occur more easily through the nailing procedure. This is why plate fixation for distal tibia fracture was preferred over IP intramedullary nail in the previous meta-analysis [26]. However, plate fixation has the disadvantage of higher soft-tissue complications and wound infections than IP nailing [25, 27, 28]. The SP intramedullary nail could be a reliable option with the combined advantages of lower anterior knee pain, a lower malalignment rate and lower soft-tissue complications.

In addition to achieving better fracture reduction quality, the SP group also required less open reduction or closed adjuvant reduction techniques during surgery than the infrapatellar group. Such differences in open reduction rates result mainly from the difficulty in controlling and maintaining the reduction of the unstable distal tibia fracture segment while keeping knee flexion in the IP approach, and, consequently, more effort (provisional plate, blocking screws, or percutaneous clamps) is needed to reduce the fracture, which increases the surgical time.

The shorter surgical time of the SP group is mainly due to the semi-extended knee position facilitating entrance identification, fracture reduction and fluoroscopic confirmation. The major advantage of the SP approach is that during the entire intramedullary nailing procedure, the affected limb is easily kept in a semi-extended position with knee flexion of 20° to 30°, rather than the hyperflexion of at least 90° in the IP approach. The difference in position increases accessibility, allowing appropriate anterior–posterior and lateral fluoroscopy images to be taken in real time. The success of nail fixation relies on obtaining the correct entry point and exit path and the quality of fracture reduction, and thus also relies on the ability to influence these factors, especially if the fluoroscopic coronal view cannot be accessed due to the flexed knee position. While Sathy et al. [29] showed a low malalignment rate (4.9%) of infrapatellar nailing with the assistance of a radiolucent tibial traction triangle in a retrospective cohort study, this device is not generally available and requires an additional transcalcaneal Steinmann pin for traction. Following cadaveric studies [30, 31], the generally accepted entry point in the coronal plane (medial side of the lateral tibial spine) changes as the tibia is rotated. Similarly, clinical studies and CT-based research [32, 33] have found that the ideal exit path of the tibial nail should be lateral to the center of the tibial plafond and talus. If the guidewire cannot be stably maintained in the correct tip position and checked with fluoroscopy during the reaming and nailing process, the nail might easily be malpositioned and fixed in malaligned reduction.

To our knowledge, this is the first systematic review on this topic to date; however, several limitations need to be addressed. These include that the enrolled studies were not prospective randomized controlled trials, there was no universal definition of the distal tibia fracture area, not all the surgical details were clearly stated in each surgical group, the outcome parameters were not all applied equally, and the follow-up time was not presented separately in three studies. More large-scale prospective randomized controlled trials with identical protocols are warranted to confirm the current findings.

## Conclusion

This study found that, for intramedullary nailing of distal tibia fractures, the suprapatellar approach showed advantages over the infrapatellar approach in higher knee functional scores, better ankle function, a lower malalignment rate, less surgical time and less need for open reduction. The suprapatellar approach may be the preferred nailing technique for treating distal tibial fractures.

## Supplementary Information

**Additional file 1: **Search strategies.

**Additional file 2: **Excluded studies, with reasons.

**Additional file 3: **Quality of the included studies assessed by the Newcastle–Ottawa scale.

## Data Availability

All data used for analysis in this study was public.
