# Supplementary material for: Suprapatellar vs infrapatellar approaches for intramedullary nailing of distal tibial fractures: a systematic review and meta-analysis
Source: J Orthop Traumatol. 2023 Apr 11;24:14. doi: 10.1186/s10195-023-00694-7 (PMC10090252; doi:10.1186/s10195-023-00694-7)
Supplement: Supplementary file 1 — Additional file 1: Search strategies. [file 10195_2023_694_MOESM1_ESM.docx]

**Appendix 1. Search strategies**

**MEDLINE (Ovid) (18th Sep 2022)**

1 exp Tibial Fractures/su [Surgery]

2 Fracture Fixation/
3 Fractures, Bone/
4 2 or 3

5 Tibia/
6 4 and 5
7 tibia$.ti.
8 fracture$.tw.
9 (metaphyseal or metaphysis or distal$).tw.

10 7 and 8 and 9
11 (1 or 6) and 9
12 10 or 11
13 Internal Fixators/ or Bone Screws/ or Fracture Fixation, Internal/ or Bone Plates/ or Bone Nails/

14 (pin$1 or nail$ or screw$1 or plate$1 or fix$ or prosthes$ or ream$ or unreamed).tw.

15 External Fixators/ or Orthopedic Fixation Devices/ or Ilizarov Technique/
16 ((Mipo adj1 technique) or (minimally adj invasive adj plate adj osteosynthesis)).tw.

17 13 or 14 or 15 or 16

18 (suprapatellar or supra or supra-patella$).tw.
19 12 and 17 and 18 [30]

2 **Embase (Elsevier)**

#1 ‘Tibia Fracture’/de or ‘Distal Tibia Fracture’/de

#2 ‘Fracture Fixation’/exp
#3 Fracture/de
#4 #2 or #3

#5 Tibia/de
#6 #4 and #5
#7 tibia*:ti.
#8 fracture*:ab,ti
#9 (metaphyseal or metaphysis or distal*):ab,ti

#10 Metaphysis/de
#11 #9 or #10
#12 #7 and #8 and #11
#13 (#1 or #6) and #11
#14 #12 or #13
#15 ‘Fixation Device’/exp
#16 ‘Surgical Equipment’/exp
#17 (pin$1 or nail* or screw$1 or plate$1 or fix* or prosthes* or ream* or unreamed):ab,ti

#18 ((Mipo NEAR/1 technique) or (minimally NEXT/1 invasive NEXT/1 plate NEXT/1 osteosynthesis)):ab,ti
#19 #15 OR #16 OR #17 OR #18
#20 #14 and #19 (232)

**3 CENTRAL (Wiley Online Library)**

#1 MeSH descriptor: [Tibial Fractures] explode all trees and with qualifier(s): [Surgery - SU]

#2 MeSH descriptor: [Fracture Fixation] this term only
#3 MeSH descriptor: [Fractures, Bone] this term only
#4 #2 or #3

#5 MeSH descriptor: [Tibia] this term only

#6 #4 and #5
#7 tibia*:ti
#8 (fracture*):ti,ab,kw

#9 (metaphyseal or metaphysis or distal*):ti,ab,kw

#10 #7 and #8 and #9
#11 #1 or #6
#12 #11 and #9

#13 #10 or #12
#14 MeSH descriptor: [Internal Fixators] this term only
#15 MeSH descriptor: [Bone Screws] this term only
#16 MeSH descriptor: [Fracture Fixation, Internal] this term only
#17 MeSH descriptor: [Bone Plates] this term only
#18 MeSH descriptor: [Bone Nails] this term only
#19 (pin* or nail* or screw* or plate* or fix* or prosthes* or ream* or unreamed):ti,ab,kw

#20 MeSH descriptor: [External Fixators] this term only
#21 MeSH descriptor: [Orthopedic Fixation Devices] this term only
#22 MeSH descriptor: [Ilizarov Technique] this term only
#23 (minimally invasive plate osteosynthesis):ti,ab,kw
#24 (Mipo near/1 technique):ti,ab,kw
#25 #14 or #15 or #16 or #17 or #18 or #19 or #20 or #21 or #22 or #23 or #24

#26 #25 and #13 (123) [Trials]

30+232+123=373 (12 duplicate) + 2 additional
