# Supplementary material for: Suprapatellar vs infrapatellar approaches for intramedullary nailing of distal tibial fractures: a systematic review and meta-analysis
Source: J Orthop Traumatol. 2023 Apr 11;24:14. doi: 10.1186/s10195-023-00694-7 (PMC10090252; doi:10.1186/s10195-023-00694-7)
Supplement: Supplementary file 2 — Additional file 2: Excluded studies, with reasons. [file 10195_2023_694_MOESM2_ESM.docx]

**Appendix 2.** Excluded studies with reasons

| **No** | **Study** | **Reference** | **Reasons for exclusion** |
| --- | --- | --- | --- |
| 1 | [Höntzsch](https://pubmed.ncbi.nlm.nih.gov/?sort=date&term=H%C3%B6ntzsch+D&cauthor_id=25410507)  2014 | Evaluation of the effectiveness of the angular stable locking system in patients with distal tibial fractures treated with intramedullary nailing: a multicenter randomized controlled trial  https://pubmed.ncbi.nlm.nih.gov/25410507/ | No planned comparison |
| 2 | Ryan 2014 | Semi-extended nailing of metaphyseal tibia fractures: alignment and incidence of postoperative knee pain  https://pubmed.ncbi.nlm.nih.gov/24751605/ | Mixed fracture location |
| 3 | Brink 2016 | Suprapatellar nailing of tibial fractures: surgical hints  https://pubmed.ncbi.nlm.nih.gov/27340503/ | Technical note |
| 4 | Çamurcu 2017 | Is talon tibial intramedullary nailing clinically superior compared to conventional locked nailing?  https://pubmed.ncbi.nlm.nih.gov/29125812/ | Not planned comparison |
| 5 | Cereijo 2018 | Intramedullary Nail Fixation of Tibial Shaft Fractures: Suprapatellar Approach  https://pubmed.ncbi.nlm.nih.gov/30588369/ | Technical note |
| 6 | [McAndrew](https://pubmed.ncbi.nlm.nih.gov/?sort=date&term=McAndrew+CM&cauthor_id=29985905) 2018 | Distal Tibial Intramedullary Nailing Using an Extraarticular, Lateral Parapatellar Approach in the Semiextended Position https://pubmed.ncbi.nlm.nih.gov/29985905/ | Technical note |
| 7 | Stengel 2018 | [Intramedullary versus extramedullary stabilization of distal extra-articular tibial fractures : The UK FixDT trial (ISRCTN99771224)] https://pubmed.ncbi.nlm.nih.gov/29947831/ | No planned comparison |
| 8 | Darnley 2020 | Tibial Intramedullary Nail From a Suprapatellar Approach in a Semi-Extended Position  https://pubmed.ncbi.nlm.nih.gov/32639353/ | Case report |
| 9 | Maslow 2020 | Radiographic Evaluation of the Tibial Intramedullary Nail Entry Point  https://pubmed.ncbi.nlm.nih.gov/32011544/ | No planned outcomes |
| 10 | Lu 2021a | Letter to the Editor: Comparison of suprapatellar versus infrapatellar approaches of intramedullary nailing for distal tibia fractures https://pubmed.ncbi.nlm.nih.gov/33509227/ | comment |
| 11 | Lu 2021b | Response to comments on: Comparison of suprapatellar versus infrapatellar approaches of intramedullary nailing for distal tibia fractures https://pubmed.ncbi.nlm.nih.gov/33546706/ | comment |
| 12 | Yang 2021 | Intramedullary nailing through suprapatellar approach with semiextended position for the treatment of tibial fractures. China J Orthop Trauma ,2021,34(5):452~457 | Not planned fracture location |
| 13 | Baker 2022 | Tibial alignment following intramedullary nailing via three approaches  https://pubmed.ncbi.nlm.nih.gov/34420095/ | No planned comparison |
| 14 | NCT01358292 | Efficacy Study of Surgical Technique in Intramedullary Tibia Nailing, Using Trigen META Tibia Nails (TrigenMETA)  NCT01358292 | Not planned fracture location |
| 15 | NCT01341418 | Suprapatellar Versus Infrapatellar Nailing in Tibial Fractures: A Pilot Study (SP-Pilot)  NCT01341418 | Not planned fracture location |
| 16 | NCT04831671 | MRI Assessment of Cartilage Damage in Tibial Nailing | Not planned fracture location |
| 17 | NCT02750072 | INfrapatellar Versus SUprapatellar Reamed Intramedullary Nailing for Fractures of the Tibia (INSURT) | Not planned fracture location |
| 18 | NCT02728362 | Suprapatellar Nailing of Tibial Shaft Fractures | Not planned fracture location |
