# Supplementary material for: Suprapatellar vs infrapatellar approaches for intramedullary nailing of distal tibial fractures: a systematic review and meta-analysis
Source: J Orthop Traumatol. 2023 Apr 11;24:14. doi: 10.1186/s10195-023-00694-7 (PMC10090252; doi:10.1186/s10195-023-00694-7)
Supplement: Supplementary file 3 — Additional file 3: Quality of the included studies assessed by the Newcastle–Ottawa scale. [file 10195_2023_694_MOESM3_ESM.docx]

**Appendix 3** Quality of included studies assessed by New-castle Ottawa scale

|  | **Selection** | | | | **Comparability** | **Outcome** | | | **Total** |
| --- | --- | --- | --- | --- | --- | --- | --- | --- | --- |
| Study  (author, year) | Representativeness of the exposed cohort | Selection of the non-exposed cohort | Ascertainment of exposure | Demonstration that outcome of interest was not present at start of study | Comparability of cases and controls on the basis of the design or analysis | Assessment of outcome | Was follow-up long enough for outcomes to occur | Adequacy of follow up of cohorts |  |
| Avilucea, 2016 | 🟑 | 🟑 | 🟑 | 🟑 | 🟑 | 🟑 | 🟑 | 🟑 | **8** |
| Lu, 2020 | 🟑 | 🟑 | 🟑 | 🟑 | 🟑 | 🟑 | 🟑 | 🟑 | **8** |
| Hague, 2021 | 🟑 | 🟑 | 🟑 | 🟑 | --- | 🟑 | 🟑 | 🟑 | **7** |
| Gao, 2022 | 🟑 | 🟑 | 🟑 | 🟑 | 🟑 | 🟑 | 🟑 | 🟑 | **8** |
